# Exosomal microRNAs as biomarkers for viral replication in tofacitinib-treated rheumatoid arthritis patients with hepatitis C

**DOI:** 10.1038/s41598-023-50963-y

**Published:** 2024-01-10

**Authors:** Tsai-Ling Liao, I-Chieh Chen, Hong-Wei Chen, Kuo-Tung Tang, Wen-Nan Huang, Yi-Hsing Chen, Yi-Ming Chen

**Affiliations:** 1 Department of Medical Research, Taichung Veterans General Hospital, Taichung, Taiwan; 2 Rong-Hsing Research Center for Translational Medicine, National Chung-Hsing University, Taichung, Taiwan; 3 Ph.D. Program in Translational Medicine, National Chung Hsing University, Taichung, Taiwan; 4 Division of Allergy, Immunology and Rheumatology, Department of Internal Medicine, Taichung Veterans General Hospital, 1650, Section 4, Taiwan Boulevard, Xitun Dist., Taichung, 407 Taiwan; 5 School of Medicine, National Yang-Ming Chiao Tung University, Taipei, Taiwan; 6 Department of Post-Baccalaureate Medicine, College of Medicine, National Chung Hsing University, Taichung, Taiwan; 7 Precision Medicine Research Center, College of Medicine, National Chung Hsing University, Taichung, Taiwan

**Keywords:** Molecular biology, Medical research, Molecular medicine

## Abstract

Notwithstanding recent advances in direct antiviral specialists (DAAs) for hepatitis C infection (HCV), it is yet a pervasive overall issue in patients with rheumatoid arthritis (RA). Exosomal microRNAs (miRNAs) is associated with HCV infection. However, it remains unknown how miRNAs respond following biologic disease-modifying antirheumatic drug (bDMARD) and targeted synthetic DMARD (tsDMARD) treatment in HCV patients with RA. We prospectively recruited RA patients taking anti-tumor necrosis factor-α (TNF-α) inhibitors rituximab (RTX) and tofacitinib. The serum hepatitis C viral load was measured using real-time quantitative reverse transcriptase PCR before and 6 months after bDMARD and tsDMARD therapy. HCV RNA replication activity was measured using an HCV-tricistronic replicon reporter system, and quantitative analysis of hsa-mir-122-5p and hsa-mir-155-5p in patients was performed using quantitative PCR. HCV RNA replication in hepatocytes was not affected by tofacitinib or TNF-α inhibitor treatment. Hsa-mir-155-5p and hsa-mir-122-5p were significantly expanded in RA patients with HCV as compared with those without HCV. We observed a dramatic increase in hsa-mir-122-5p and a decrease in hsa-mir-155-5p expression levels in patients taking RTX in comparison with other treatments. Finally, a reduction in hsa-mir-122-5p and an increase in hsa-mir-155-5p were observed in a time-dependent manner after tofacitinib and DAA therapy in RA-HCV patients. These results showed that hsa-mir-155-5p and hsa-mir-122-5p were significantly increased in RA-HCV patients as compared with those without HCV after taking tofacitinib. Hsa-mir-155-5p and hsa-mir-122-5p may be potential biomarkers for treatment efficacy in RA patients with HCV.

## Introduction

An expanding body of evidence indicates that targeted treatment can improve personal satisfaction and quality of life in rheumatoid arthritis (RA) patients^1,2^. A major issue in treating patients is that rheumatologists should initiate immunosuppression, especially in those with concomitant chronic hepatitis, particularly hepatitis C infection (HCV). Despite the progress made in direct antiviral specialists (DAAs) for HCV over the last ten years, it is yet a pervasive overall issue in RA patients with rheumatoid arthritis^1,2^. Our previous study showed that the utilization of tumor necrosis factor (TNF)-α in RA patients with HCV did not appear to influence HCV replication^3^. However, the HCV viral burden expanded after rituximab (RTX) treatment. HCV viral replication might react distinctively to biologic disease-modifying antirheumatic drugs (bDMARDs) and designated engineered DMARDs (tsDMARDs) with various systems of activity in RA patients. Therefore, there is much interest in identifying biomarkers that will facilitate identification of whether a patient will react diversely to bDMARDs and tsDMARDs in terms of concomitant HCV infection.

Short-term treatment with tocilizumab, an IL-6 receptor-targeted monoclonal antibody, has been reported to have no effect on the viral load of HCV in RA-HCV patients^1,2^. Abatacept, binds CTLA-4 to block the CD28-CD80/CD86 co-stimulatory pathways, has been demonstrated without safety alerts in RA patients with HCV^3^. Our recent study revealed that tofacitinib, a Janus kinase (JAK) inhibitor, did not interfere with HCV replication in RA patients^4^. Considering potential safety issues following HCV viral replication, it is crucial to identify biomarkers of host immune responses to targeted therapies to achieve the best outcomes in patients with RA.

Recently, microRNAs (miRNAs) have emerged as another category of biomarkers, and patients with RA may exhibit alternation of miRNAs expression^5,6^. These are a bountiful class of endogenous, short noncoding, RNA that controls the translation of messenger RNAs (mRNAs). MiRNAs have an average of ~ 22-nucleotides, which intervene in mRNA cleavage, translational suppression, or mRNA destabilization^7^. Over 2,000 human miRNAs have now been discovered and recorded (miRBase Delivery 20.0)^8^. Individual miRNAs as molecular biomarkers and therapeutic targets for diagnosis and prognosis are well-documented in cancers^9^. In addition to regulating the expressions of genes at the post-transcriptional and translational levels, miRNAs have likewise been implicated in modulation of the host immune response in HCV infection^5,6^. Has-miRNA-155-5p is upregulated in RA-HCV patients and is predominantly expressed in B cells. A study indicated that macrophages could secrete innate anti-HCV factors to hepatocytes infected by HCV through exosomes^7^. Exosomes are membrane vesicles released from donor cells (e.g., B cells) that are considered to be a new mode of cell–cell communication and may carry cellular components to recipient cells (e.g., hepatocytes)^10^. We speculated that the impact of RTX on reactions to B cells might have a potential effect on the creation and transmission of anti-HCV factors (e.g., has-miRNA-155-5p) through exosomes, and in this way to alter HCV replication.

MiRNA-122, the most abundant microRNA in hepatocytes, plays a crucial role in maintaining hepatocyte function^11^. Functioning as a tumor suppressor, miR-122 inhibits HBV replication, while HBV infection downregulates miR-122 expression^12–14^. Recently, some reports have revealed that miRNA-122 can be utilized as a marker for hepatic diseases and HBV infections^15–17^. A combination of these experimental results has been applied to rat models with hepatic disease, revealing that changes in plasma concentration of miRNA-122 may be indicative of the severity of the disease in both rat and human models. Notably, changes in miRNA-122 levels appear to precede those in aminotransferase activities, indicating its potential as an early blood marker for hepatic diseases, including HBV^17^. In this context, researchers investigated the association between has-miRNA-122 and HBV infection, demonstrating that serum levels of has-miRNA-122 could effectively differentiate between healthy individuals and those infected with HBV^15,18^.

In our study, we aimed to investigate the potential effects of both bDMARDs and tsDMARDs on HCV replication in hepatocytes and assessed their impact on the expression levels of hsa-mir-155-5p and hsa-mir-122-5p in hepatocytes and B cells. Additionally, we examined the expression levels of hsa-mir-155-5p and hsa-mir-122-5p in peripheral blood mononuclear cells (PBMCs) and assessed these levels in serum of RA patients with HCV infection who were treated with bDMARDs and tsDMARDs.

## Methods

### Study participants

Overall, 45 participants were enrolled, including 25 RA-HCV patients, 10 RA patients and 10 controls (the control group did not have HCV or RA). Among the 25 RA-HCV patients, 6tofacitinib-treated, 8 RTX-treated, 5 TNFi-treated, and 6 patients with non-biologic therapy were recruited from Taichung Veterans General Hospital, Taiwan. All participants had persistently active disease as defined by a Disease Activity Score in 28 joints (DAS28) > 5.1^8^ after treatment with conventional synthetic disease-modifying antirheumatic drugs (csDMARDs); thus, biologic therapy or tsDMARD therapy based on the British Society for Rheumatology guidelines was administered^19^. RA patients received anti-TNF-α therapy with adalimumab (40 mg subcutaneously every other week). The RTX-treated RA patients were prescribed intravenous RTX (1000 mg twice with a 14-day interval at 6-month intervals for 1 year). Other patients received tofacitinib (5 mg twice a day). The serum HCV viral load was measured using real-time quantitative reverse transcriptase PCR before and 6 months after bDMARD and tsDMARD therapy. The study protocol was conducted in accordance with the Declaration of Helsinki and approved by the Ethics Committee of Taichung Veterans General Hospital’s Institutional Review Board (IRB No. CE19138B), and all of the participants gave written informed consent.

### Cell culture and HCV replication

Huh-7 and HCV-tricistronic replicon cells were grown in Dulbecco’s modified Eagle’s medium (DMEM) supplemented with 2% human serum, 100 units/ml penicillin, and 100 mg/ml streptomycin at 37 °C, with 5% CO_2_. For the exosome study, cells were cultured in media supplemented with 10% exosome-depleted fetal bovine serum (FBS; Thermo Fisher Scientific, USA) after biologic treatment. PBMCs were immediately sampled from peripheral venous blood using Ficoll-Paque™ PLUS (GE Healthcare Biosciences, Uppsala, Sweden) density gradient centrifugation.

Total cellular RNA was extracted from PBMCs by using TRIzol Reagent (Thermo Fisher Scientific, USA) according to the manufacturer’s instructions, and further quantified by spectrophotometry at 260 nm and 280nm.

In order to estimate the impact of tofacitinib on HCV RNA replication, we inspected HCV replication activity by utilizing an HCV-tricistronic replicon reporter system^8,20^ to assay viral RNA replication activity. HCV replicon cells were treated with individual biologics (10 μg/ml) for 72 h. Then, cell viability assays were performed, and HCV RNA replication activity was estimated utilizing a luciferase assay.

### Exosome isolation and quantification

Exosome isolation kits were used to collect exosomes according to the manufacturer’s instructions. In brief, samples were centrifuged at 2,500 rpm for 10 min at 4°C, and then cell debris was removed through a 0.22-μm filter. Exo Quick exosome precipitation solution (System Biosciences, USA) was used to extract the serum- and Huh7.5 cell-derived exosomes. Then, the purified exosomes were verified by immunoblotting. Exosomes were further quantified by using ExoELISA-ULTRA Complete Kit (EXEL-ULTRA-CD63-1, System Biosciences, USA) to quantify the exosomes surface marker CD63 according to the manufacturer’s instructions.

### Exosomal miRNA quantitative PCR

Total RNA extraction was performed using TRIzol® Reagent (Thermo Fisher Scientific) and purified using a RNeasy MinElute^®^ Cleanup Kit (QIAGEN, Germany) as previously reported^21^. Purified total RNAs were quantified using an ND-1000 spectrophotometer (Thermo Fisher Scientific). Reverse transcription was carried out using a StepOnePlus™ Real-Time PCR System (Thermo Fisher Scientific). The expression levels of miRNAs were quantified using a TaqMan MicroRNA assay kit (Thermo Fisher Scientific). Twenty-five femtomoles of synthetic Caenorhabditis elegans miRNA (cel-miR-39, Thermo Fisher Scientific, USA) were added to each sample as the internal control. The miRNA expression was quantified using the TaqMan MicroRNA assays kit (Thermo Fisher Scientific, USA) according to the manufacturer’s protocol. QRT-PCR reactions were performed on the StepOnePlus Real-Time PCR System (Thermo Fisher Scientific, USA) using a standard protocol (95°C 10min 1cycle; 95°C 15s, 60°C, 1min, 40 cycles). Each sample were run in triplicate. The fold expression of the target gene relative to the averaged internal control gene in each sample will be calculated using the comparative threshold cycle (Ct) method and evaluated by 2^−△△Ct^, △△Ct = Patient (Ct_miRNAs gene_ − Ct_cel-miR-39_) − Mean of controls (Ct_miRNAs gene_ − Ct_cel-miR-39_).

### Immunoblotting

The exosomes were lysed in RIPA buffer (25mM Tris–HCl pH 7.6, 150mM NaCl, 1% NP-40, 1% sodium deoxycholate, and 0.1% SDS) containing a protease inhibitor cocktail (Complete, Roche, Germany). Twenty micrograms of total protein from exosomes lysate were loaded and separated on a standard sodium dodecyl sulfate–polyacrylamide gel electrophoresis (SDS-PAGE) gel and were transferred to a polyvinylidene difluoride membrane (Millipore, USA). The membranes were incubated with primary antibodies (e.g., CD63, CD81, or CD9) and peroxidase-conjugated secondary antibodies (goat anti-rabbit at 1:20,000, goat anti-mouse at 1:10,000). The results were detected by using Amersham TM Imager 680 (GE Healthcare Life Sciences, USA) after membrane incubation with enhanced chemiluminescence substrates (Millipore, USA).

### Statistical analysis

An unpaired, two-tailed Student’s t-test was used for between-group comparisons. One-way analysis of variance (ANOVA) with the Bonferroni post hoc test was used for multiple comparisons. HCV viral loads following bDMARD and tsDMARD therapy were analyzed by the Kruskal–Wallis test. Comparisons of hsa-mir-155-5p and hsa-mir-122-5p after treatment with bDMARDs and tsDMARDs were performed using the Mann–Whitney U test. The dynamics of HCV viral loads at different time points were compared by the Wilcoxon signed-rank test. Statistical significance was defined as p values < 0.05, and all analyses were conducted using GraphPad Prism 8 and SAS version 9.4 software (SAS Institute Inc., Cary, NC).

## Results

### Characteristic of participants

**Figure 1:**
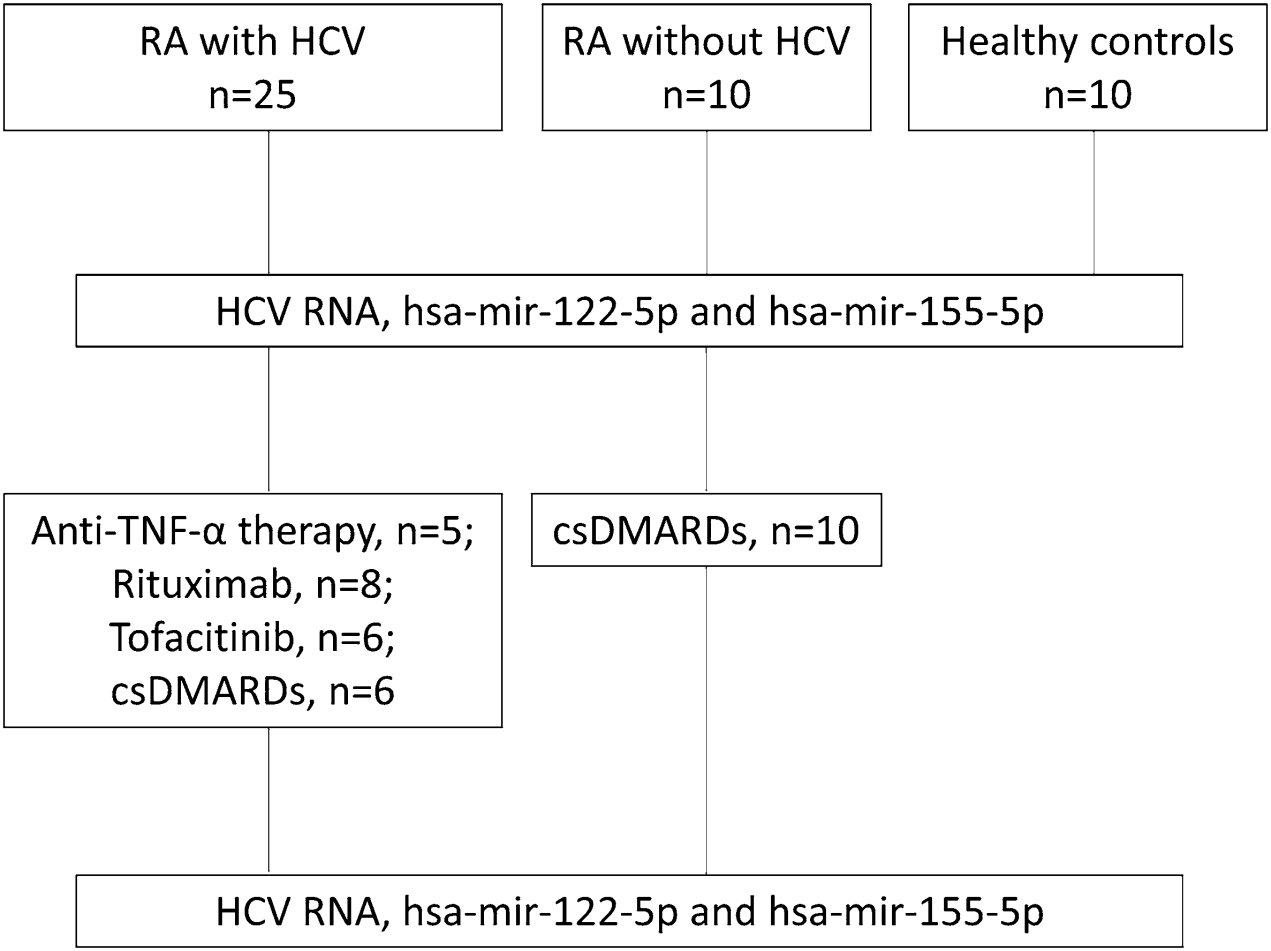
Illustrative flow chart of the study design.

This study involved 45 participants, comprising 25 RA-HCV patients, 10 RA patients, and 10 controls (Fig. 1). Among the 25 RA-HCV patients, there were 6 treated with tofacitinib, 8 with rituximab (RTX), 5 with TNF inhibitors (TNFi), and 6 with non-biologic therapy. The serum HCV viral load, along with the expression levels of hsa-mir-122-5p and hsa-mir-155-5p, was measured in all participants both before and six months after various therapeutic interventions. Table 1 displays the basic characteristics of the participants. The mean ages of the 25 RA-HCV patients in the TNFi-treated, RTX-treated, tofacitinib-treated, and csDMARDs groups were 60.7 ± 10.7 years, 63.5 ± 8.8 years, 57.5 ± 10.9 years, and 52.9 ± 9.6 years, respectively. Higher HCV viral load was observed in the HCV-positive RA patients. Within each group (RTX-treated, tofacitinib-treated, and csDMARDs), one participant displayed liver fibrosis grading exceeding III or IV (%).

**Table 1:** Demographic data of enrolled participants.

	RA with HCV				RA without HCV	Healthy controls n = 10
	Anti-TNF-α therapy n = 5	Rituximab n = 8	Tofacitinib n = 6	csDMADRs n = 6	csDMADRs n = 10	
Age	60.7 ± 10.7	63.5 ± 8.8	57.5 ± 10.9	52.9 ± 9.6	60.7 ± 9.8	60.5 ± 9.9
Gender (female, %)	4 (80.0)	6 (75.0)	5 (83.3)	5 (83.3)	7 (70.0)	8 (80.0)
DAS28	5.6 ± 0.9	5.7 ± 1.0	5.3 ± 0.9	5.5 ± 1.0	4.4 ± 0.9	
Baseline HCV viral load (IU/ml)	2.2 × 10^6^	2.7 × 10^6^	2.6 × 10^6^	1.9 × 10^6^		
Liver fibrosis grading > III or IV (%)	0 (0)	1 (12.5)	1 (16.6)	1(16.6)		
Concomitant MTX (%)	3 (60.0)	6 (75.0)	4 (66.7)	6 (100.0)	5 (50.0)	

RA, rheumatoid arthritis, HCV, hepatitis C; csDMARDs, conventional synthetic disease-modifying antirheumatic drugs; anti-TNF-α therapy, anti-tumor necrosis factor-α; MTX, methotrexate.

### Effect of Tofacitinib, TNF-α Inhibitors, and Rituximab on HCV Viremia in RA Patients

**Figure 2:**
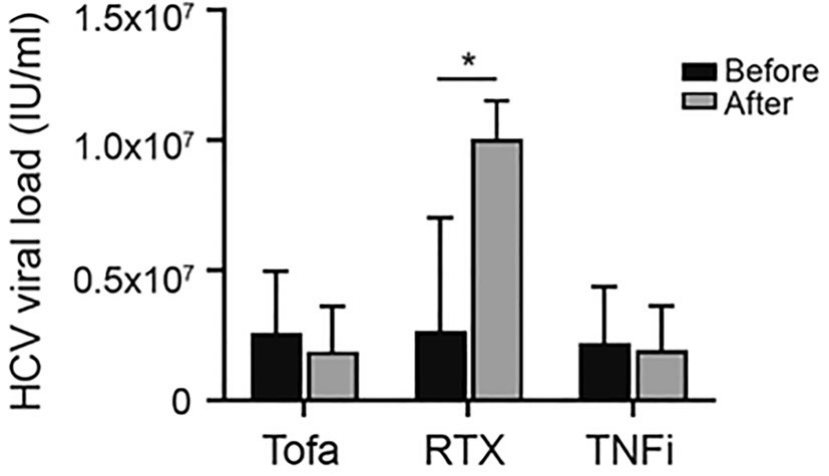
Comparison of HCV viral load in RA patients receiving different treatments. Tofa, tofacitinib; RTX, rituximab; TNFi, tumor necrosis factor-α inhibitor; HCV, hepatitis C virus. Data are presented as the mean ± SD. *P < 0.05.

Previously, RTX was shown to increase the HCV viral load to a greater extent than TNF-α inhibitors^21^. In this study, we utilized the HCV viral load test to assess whether tofacitinib influences HCV viremia in HCV-positive RA patients receiving anti-TNF-α, RTX and tofacitinib. As shown in Fig. 2, a significantly increased HCV viral load was found in patients after receiving RTX treatment (before versus after: 2.7 × 10^6^ vs. 1.0 × 10^7^ IU/ml, *P* < 0.05), but there was no significant difference in HCV viral load in patients after treatment with tofacitinib (2.6 × 10^6^ vs. 1.9 × 10^6^ IU/ml, *P* > 0.05) or TNF-α inhibitor (2.2 × 10^6^ vs. 1.9 × 10^6^ IU/ml, *P* > 0.05).

### Tofacitinib does not directly impact HCV replication

**Figure 3:**
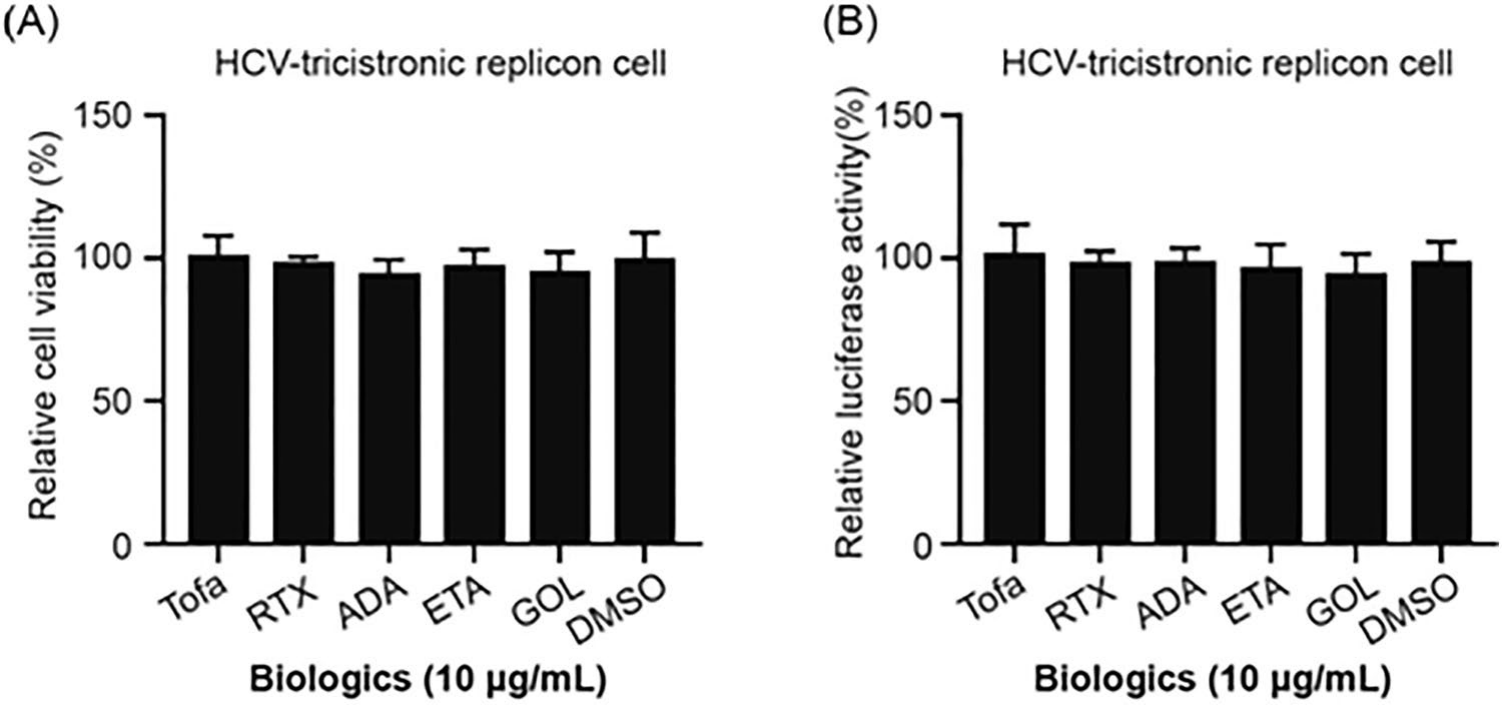
Expression of cell viability (**A**) and HCV genome replication (**B**) in hepatocytes treated with different biologics. Tofa, tofacitinib; RTX, rituximab; ADA, adalimumab; ETA, etanercept; GOL, golimumab; HCV, hepatitis C virus; DMSO, 2% dimethyl sulfoxide. All experiments were performed in triplicate, and data are presented as the mean ± SD.

To assess the influence of tofacitinib on HCV RNA replication, we examined HCV replication activity using an HCV-tricistronic replicon reporter system^8^. As shown in Fig. 3, there was no significant difference in cell viability (Fig. 3A) or HCV genome replication (Fig. 3B) between HCV replicon cells treated with tofacitinib or other biologics (including rituximab, adalimumab, etanercept, and golimumab) and DMSO control cells. These results indicated that tofacitinib and other biologics had no hepatotoxic effect and did not directly impact HCV replication in hepatocytes.

### Hsa-mir-122-5p and hsa-mir-155-5p may be used as potential biomarkers to monitor HCV viremia status in RA patients during antirheumatic therapy

To validate the purity of exosomes used in this study, we measured particle size of extracellular vesicles (EVs) (Zetasizer Nano ZS90, Malvern Instruments, Malvern, UK). The result proving the average particle size of EVs we extracted is 197.6 nm, consistent with the size range of typical exosomes from 30 to 200 nm (Supplementary Fig. S1A). In addition, these EVs expressed specific exosomes markers (Supplementary Fig. S1B). Elevated exosomes levels in the serum of RA patients with HCV infection (7.48 ± 1.51 × 10^9^ particles/ml, *P* < 0.005, Supplementary Fig. S1C) compared with those without infection (5.14 ± 0.85 × 10^9^ particles/ml) or healthy control (4.91 ± 0.94 × 10^9^ particles/ml). Among RA patients with HCV infection, there was no significantly difference on the levels of exosomes in those receiving tofacitinib therapy (6.45 ± 0.81 × 10^9^ particles/ml).

Previously, we demonstrated that the circulating hsa-mir-155-5p level was negatively correlated with HCV viral load and was associated with RTX-related HCV activity enhancement in RA patients; hsa-mir-155-5p may therefore be a potential diagnostic biomarker^21^. As observed in a previous study, an increased expression level of hsa-mir-122-5p was recorded in serum samples of patients with HCV infection as compared with that in controls^19^. Additionally, an increased hsa-mir-122-5p (1.85 ± 0.10-fold, *P* < 0.005) level was detected in Huh7.5 cell-derived exosomes after HCV infection^19^. Our results suggested that hsa-mir-122-5p may be a potential biomarker to assess whether patients have HCV infection.

### Differential expression of hsa-mir-122-5p and hsa-mir-155-5p in patients undergoing varied antirheumatic therapies

**Figure 4:**
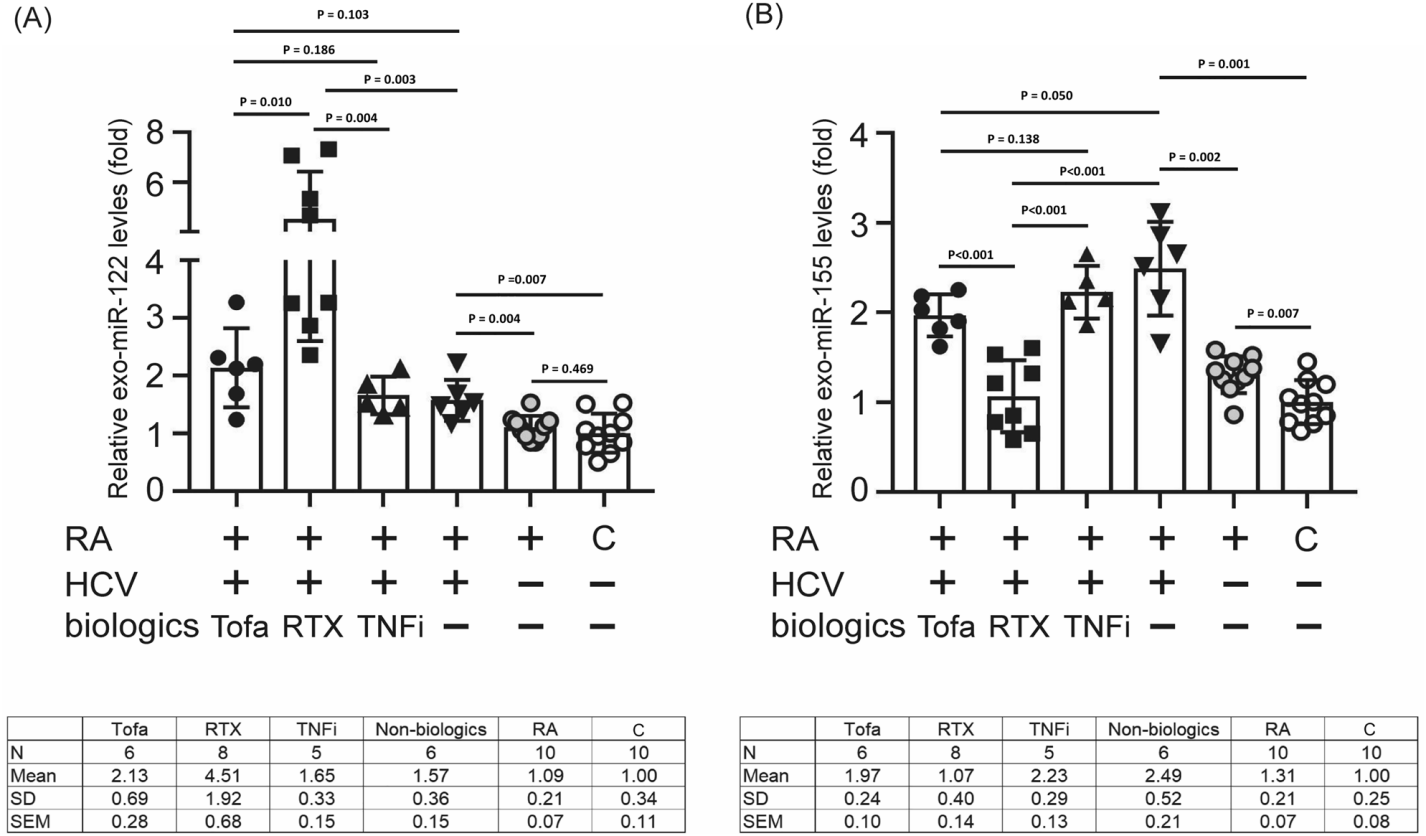
Expression levels of has-miR-122-5p (**A**) and has-miR-155-5p (**B**) in RA patients receiving different biologics. All experiments were performed in triplicate, and data are presented as the mean ± SD. Tofa, tofacitinib; RTX, rituximab; TNFi, tumor necrosis factor-α inhibitor; HCV, hepatitis C virus; RA, rheumatoid arthritis. P-values are utilized to assess statistical significance through the application of Student’s t-test.

To further evaluate whether hsa-mir-122-5p and hsa-mir-155-5p could be used as biomarkers for monitoring HCV viremia status in HCV patients during biological therapy, we analyzed the expressions of hsa-mir-122-5p and hsa-mir-155-5p in patients receiving different antirheumatic therapies. Overall, 45 participants were enrolled, including 25 RA-HCV patients, 10 RA patients and 10 controls. Among the 25 RA-HCV patients, 6 were tofacitinib-treated, 8RTX-treated, 5 TNFi-treated, and 6 patients received non-biologic therapy. As shown in Fig. 4, compared with the controls, the expression levels of hsa-mir-122-5p and hsa-mir-155-5p in non-HCV RA group were increased 1.09 ± 0.21-fold (Fig. 4A) and 1.57 ± 0.36-fold (Fig. 4B), respectively. Among the RA patients with HCV, the hsa-mir-122-5p expression level was dramatically increased in patients treated with RTX (4.51 ± 1.92-fold) or TNF-α inhibitor (1.65 ± 0.33-fold) as compared with those receiving non-biologic agents. A slightly increased hsa-mir-122-5p level was observed in the patients who received tofacitinib therapy (2.13 ± 0.69-fold), but the difference was not significant. Compared to the control group, the expression level of hsa-mir-155-5p was significantly increased in RA patients with HCV (2.49 ± 0.52-fold vs. 1.0 ± 0.25-fold, p = 0.001). In comparison to RA alone, the hsa-mir-155-5p expression level was significantly elevated in RA patients with HCV (2.49 ± 0.52-fold vs. 1.31 ± 0.21-fold, p = 0.002) (Fig. 4B). Notably, a significantly greater decrease in the hsa-mir-155-5p expression level was observed in patients after RTX therapy (1.07 ± 0.40-fold), TNF-α inhibitor treatment (2.23 ± 0.29-fold, *P* < 0.001), or tofacitinib treatment (1.97 ± 0.24-fold, *P* < 0.001) than in those receiving non-biologic agents (2.49 ± 0.52-fold, *P* < 0.001). However, there were no significant differences in the expression levels of both hsa-mir-122-5p and hsa-mir-155-5p in patients under tofacitinib therapy as compared with those receiving non-biologic agents or TNF-α inhibitor treatment. This result suggested that tofacitinib may not affect miR-155-5p production, which could inhibit HCV replication.

**Figure 5:**
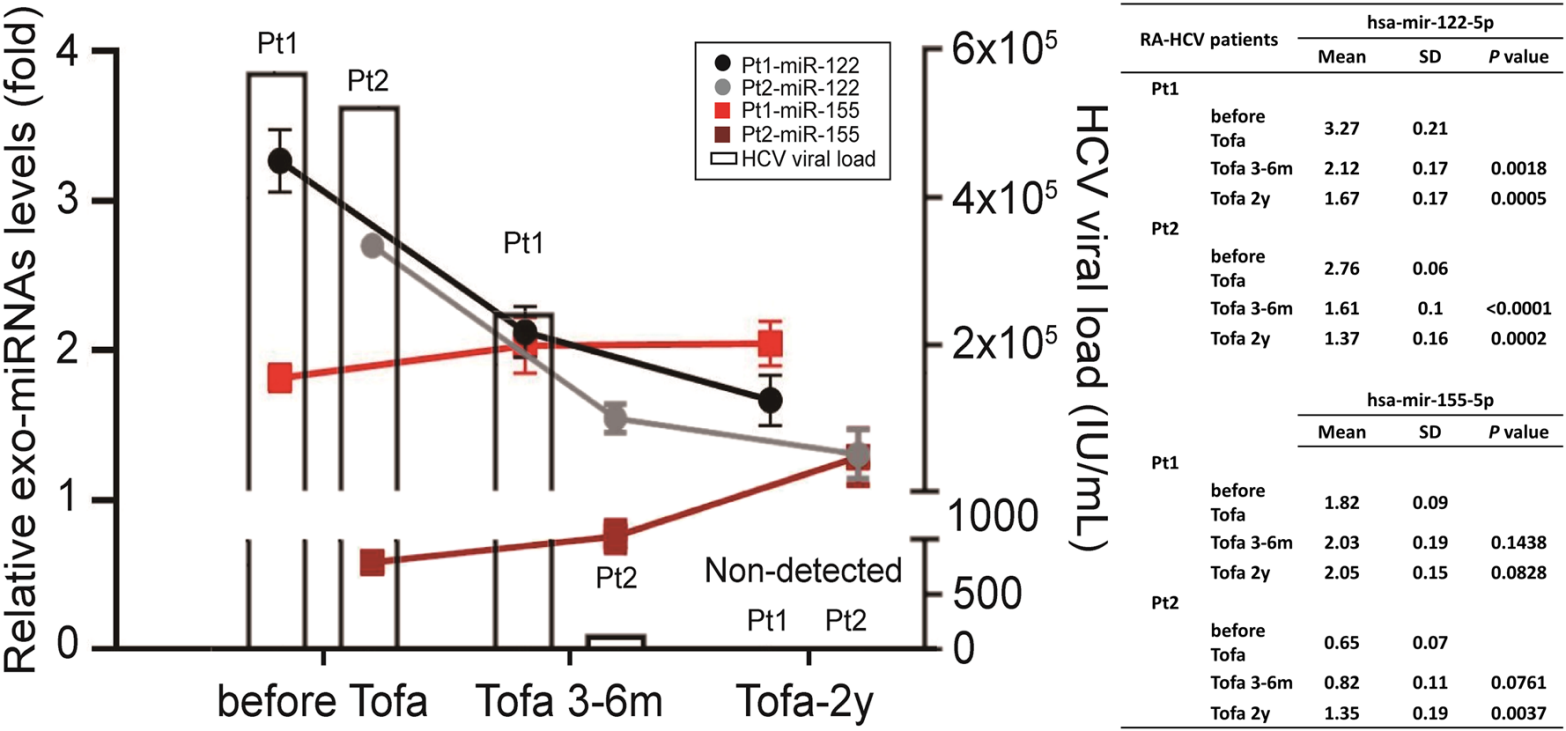
Dynamics of HCV viral load, and hsa-mir-122-5p and hsa-mir-155-5p levels in the serum of two RA patient during tofacitinib (Tofa) therapy and direct-acting anti-viral therapy for HCV. All experiments were performed in triplicate, and data are presented as the mean ± SD. P-values are utilized to assess statistical significance through the application of Student's t-test. Pt1, patient 1; Pt2, patient 2.

Finally, we observed the dynamics of HCV viral load and the hsa-mir-122-5p and hsa-mir-155-5p expression levels in the serum of two RA-HCV patients during the tofacitinib therapy period. We found that a significant decrease in hsa-mir-122-5p in the tofacitinib-treated patient was time-dependent (Fig. 5) compared with another RA patient without HCV infection, as the HCV viral load decreased simultaneously after direct-acting antiviral therapy for HCV. Following 3–6 months of Tofacitinib (Tofa) therapy, the expression of hsa-mir-122-5p exhibited a significant decrease. This decrease persisted after 2 years of continued Tofa use, with fold changes of 2.12 ± 0.17 and 1.67 ± 0.17 (p = 0.0018 and p = 0.0005, respectively) of Patient 1 (Pt1) and 1.61 ± 0.1 and 1.37 ± 0.16 (p < 0.0001 and p = 0.0002, respectively) of Patient 2 (Pt2). Furthermore, we observed a slight but significant elevation in hsa-mir-155-5p expression for Pt2 (1.35 ± 0.19-fold, p = 0.0037) compared to an RA patient without HCV infection, after 2 years of continued Tofa use.

## Discussion

This study demonstrated that HCV RNA replication in hepatocytes was not affected by tofacitinib or TNF-α inhibitor treatment. Our results also showed that RTX increased the HCV viral load to a more noteworthy degree than TNF-α inhibitor therapy in RA-HCV patients. Compared with non-HCV RA patients, the expression level of hsa-mir-122-5p was increased in RA-HCV patients. The microRNAs hsa-mir-155 and hsa-mir-122 may be potential biomarkers for assessing RA patients with HCV infection. Furthermore, there was a marked increase in hsa-mir-122-5p and a decrease in hsa-mir-155-5p in RA-HCV patients taking RTX as compared with the conventional synthetic DMARD group. Rituximab is a biologic agent for the treatment of refractory RA that causes B cell depletion as a therapy for patients who fail to respond to other biologics^22^. Recently, one randomized controlled trial demonstrated that selective depletion of B cells with the use of RTX led to sustained clinical improvement inpatients with RA^23^. An old age and a long duration of RTX therapy were characteristics of our RA-HCV patients^21^. Our results showed that B cell-derived hsa-mir-155-5p is essential in RTX-related HCV viral replication activity. Recently, a study indicated that the exosome concentration is inversely correlated with advancing age, which is associated with biologic dysfunction in B cells^24^. Therefore, we proposed that aging and long-term RTX treatment might influence the biological functions of B cells and cause diminished exosome levels and a lowered hsa-mir-155-5p expression, leading to increased HCV viremia.

Exosomes are extracellular vesicles delivered by cells that convey proteins, lipids, and nucleic acids, and have the capacity for intercellular communication^25^. MiRNAs can be coupled into exosomes and carried by exosomes to aid biological functions^25^. A number of studies have demonstrated that human immune cells are capable of recognizing virus-infected cells and produce exosomal miRNAs in response to viral infection^26–28^. In a cell model study, in pigment epithelial cells, B cell-derived miR-155 regulated biological function through exosome transmission^29^. Moreover, Zhang et al.^30^ showed that miR-155 is increased in the hepatocytes of patients with chronic HCV. Jiang et al.^31^ demonstrated that miR-155 is a critical mediator of anti-inflammatory activity in hepatocytes that control innate and adaptive immune responses during HCV. Several studies have investigated whether miR-155 increases the expressions of pro-inflammatory cytokines (e.g., TNF-α, interleukin-6, interferon-sensitive genes^31–33^), which are associated with the repression of HCV production^34,35^. MiRNA-155 is one of the miRNAs induced during immune activation and profoundly regulates the innate immune response^36–38^; its transcription is induced by the involvement of Toll-like receptors (TLRs) and inflammatory cytokines, or upon viral infection^36–38^. According to previous studies, miR-155 acts as a negative mediator of the innate immune response^39,40^, and adaptor proteins MyD88 and IKKε were recognized as potential targets of miR-155^39,40^. Another study confirmed that miR-155 transgenic mice produced higher levels of TNF-α in response to lipopolysaccharide^41^. Concordantly, miR-155 is significantly upregulated in synovial fibroblasts and synovium from inflamed joints of patients with RA, and its expression can be further induced by TNF-α, IL-1β, and ligands of TLR2 through TLR4^42^. This finding demonstrated that the inflammatory milieu might be responsible for the changed expression of miR-155 in these cells^41,42^.This was consistent with the report of Blumlet al, who demonstrated that miR-155 knockout mice did not develop collagen-induced arthritis where a prominently lower production of many proinflammatory cytokines was noted^43^.

In this study, we analyzed the hsa-mir-122-5p expression levels in RA-HCV patients receiving different antirheumatic therapies. Among RA patients with HCV, hsa-mir-122-5p was dramatically increased in patients treated with RTX (4.51 ± 1.92-fold, Fig. 3A), and the HCV viral load increased simultaneously (Fig. 1), as compared with those receiving non-biologic agents (1.57 ± 0.36-fold) or TNF-α inhibitors (1.65 ± 0.33-fold), as shown in Fig. 3A. This finding was consistent with a previous study reporting elevated levels of hsa-mir-122-5p in the sera of patients with HCV as compared with those in controls^19^. Additionally, an increased hsa-mir-122-5p level (1.85 ± 0.10-fold, *P* < 0.005) was detected in Huh7.5 cell-derived exosomes after HCV infection^19^. Thus, further identification of the function of extracellular hsa-mir-122-5p will clarify its role in RA-HCV patients and may have important implications for the treatment of HCV.

MiRNA-122 is the most abundant microRNA in hepatocytes and plays an important role in the maintenance of hepatocyte function^11^. However, the roles of miR-122 in infections of hepatitis viruses are inconsistent. As a tumor suppressor, miR-122 inhibits HBV replication, and HBV infection in turn downregulates miR-122 expression^12–14^. Interestingly, through co-operation with the 5′-end of HCV genomic RNA, miR-122 is essential for the replication of the hepatitis C virus^6,44^. In addition, miR-122 may regulate interferon (INF) signaling by targeting suppressor of cytokine signaling 3 (SOCS3) or SOCS1^45,46^ to repress the activation of INFs in response to different viral nucleic acids, especially HCV RNA^47^. In the mechanism, miRNA-22 downregulates the phosphorylation (Tyr705) of STAT3, thereby removing the negative regulation of STAT3 on IFN signaling. Furthermore, miR-122 plays a critical immunomodulatory role in hepatocytes through a miR-122-RTKs/STAT3-IRF1-IFNs regulatory circuitry, and acts as a significant membrane in modulating hepatocyte innate immunity^47^. In a human liver organoid model, miRNA-122 expression contributed to inflammation resolution via TNF-α and IL-6 suppression^48^. In addition, Valentina Manfe et al. identified that miRNA-122 may play a role in the enhanced expressions of inflammatory factors through Akt activation^49^. Although a possible role of miRNA-122 in the regulation of inflammatory cascades has been well-described, the exact mechanism through which miRNA-122 affects the TNF-α and IL-6 levels and the consequent link to RA patients remain elusive. Future investigations are needed to clarify the underlying mechanisms between miRNA-122 and IL-6 and to determine their potential associations, and to identify the key role of miRNA-122 in the presence of chronic inflammation in patients with RA.

In recent years, the use of biologic agents and small-molecule target drugs has been proven to be effective for some immune inflammatory diseases that do not respond well to traditional drugs. Tofacitinib is a small-molecule oral-dosage form of the JAK inhibitor, which can inhibit cell inflammation. Our study revealed that tofacitinib has no effect on HCV viremia in RA patients. The tolerability profile of tofacitinib is generally similar to that of bDMARDs. There were no notable differences in cell viability (Fig. 2A) or HCV genome replication (Fig. 2B) between tofacitinib- or other biologics-treated HCV replicon cells. Slightly increased hsa-mir-122-5p and hsa-mir-155-5p levels were observed in patients who received tofacitinib therapy (2.13 ± 0.69-fold and 1.97 ± 0.24-fold, respectively). We examined the dynamics of the HCV viral load and the hsa-mir-122-5p and hsa-mir-155-5p expression levels in the serum of one RA-HCV patient during the tofacitinib therapy period. Our results demonstrated a significant decrease in exomiR-122 expression and a slight increase in hsa-mir-155-5p expression in the tofacitinib-treated patient in a time-dependent manner (Fig. 4). This result suggested that tofacitinib may not affect hsa-mir-155-5p production, which could inhibit HCV replication. Further experiments are required to confirm our study results.

Despite that our study makes significant contributions by demonstrating the differential expression of hsa-mir-122-5p and hsa-mir-155-5p in response to RTX and tofacitinib treatment in RA-HCV patients, this study does not provide data to establish a definitive temporal sequence between the elevation of miRNAs and the increase in HCV viral load. This indeed represents a limitation of our current research. Future research should focus on a longitudinal study design with more frequent sampling intervals. Such a study would be invaluable in elucidating the temporal dynamics of miRNA expression in relation to changes in HCV viral load, thereby providing deeper insights into the early host responses to viral replication and the potential predictive value of these miRNAs. This future direction could significantly advance our understanding of the interplay between RA, HCV, and treatment response, ultimately contributing to improved patient management and treatment strategies.

In summary, we demonstrated that HCV RNA replication in hepatocytes was not affected by tofacitinib treatment. Hsa-mir-155-5p and hsa-mir-122-5p were significantly increased in RA-HCV patients as compared with those without HCV after taking tofacitinib. Hsa-mir-155-5p and hsa-mir-122-5p may be potential biomarkers for RA patients with HCV infection following advanced therapy. Further studies are needed to confirm our findings.

### Supplementary Information

Supplementary Figure S1.

Supplementary Figure S2.

## Data Availability

All data are generated or analyzed are included in this study.
