# Supplementary material for: Exosomal microRNAs as biomarkers for viral replication in tofacitinib-treated rheumatoid arthritis patients with hepatitis C
Source: Sci Rep. 2024 Jan 10;14:937. doi: 10.1038/s41598-023-50963-y (PMC10776842; doi:10.1038/s41598-023-50963-y)
Supplement: Supplementary file 1 — Supplementary Figure S1. [file 41598_2023_50963_MOESM1_ESM.pdf]

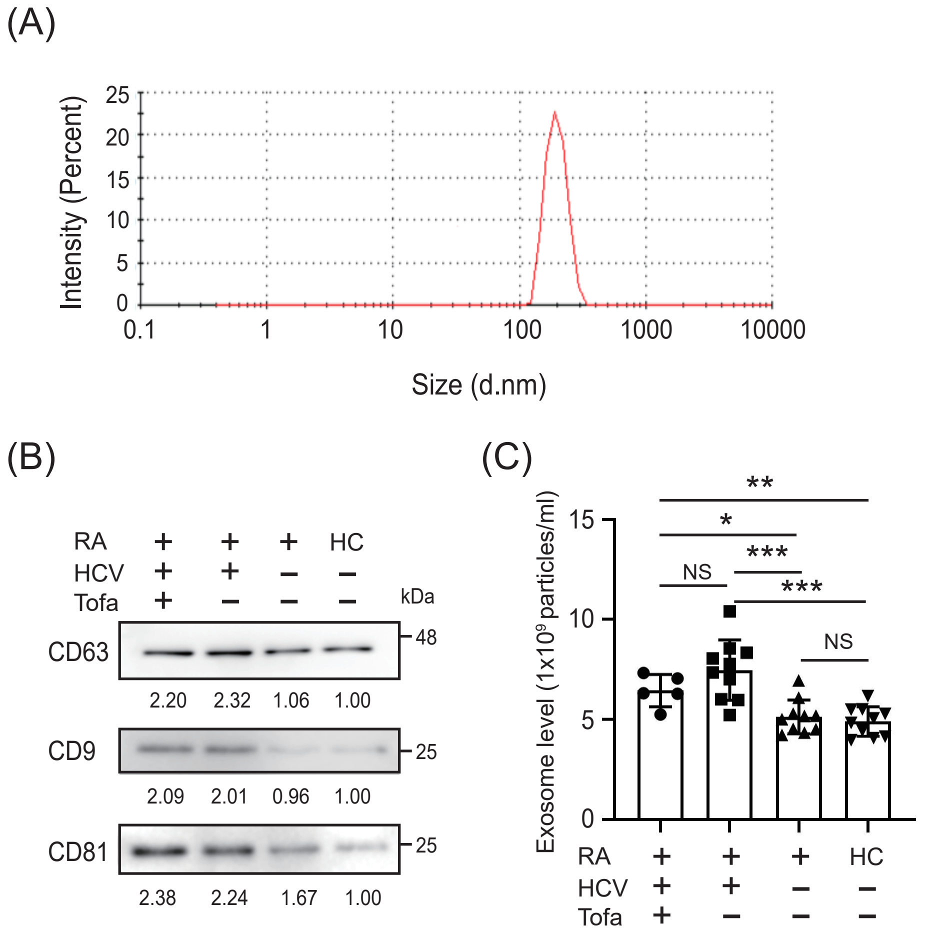

Supplementary Figure S1 (A) The particle size of extracellular vesicles were extracted from sera of rheumatoid arthritis (RA) patient with HCV infection. (B) Expression of exosomal protein CD63, CD9, and CD81 and (C) concentration of exosomes in sera from RA patient with HCV infection receiving tofacitinib (Tofa) therapy, RA patient with HCV infection, RA patient, and healthy control (HC). *P<0.05,**P<0.01,***P<0.005 was determined by using student's t-test. NS, not significant.
