# Supplementary figures and images for: Exosomal microRNAs as biomarkers for viral replication in tofacitinib-treated rheumatoid arthritis patients with hepatitis C
Source: Sci Rep. 2024 Jan 10;14:937. doi: 10.1038/s41598-023-50963-y (PMC10776842; doi:10.1038/s41598-023-50963-y)

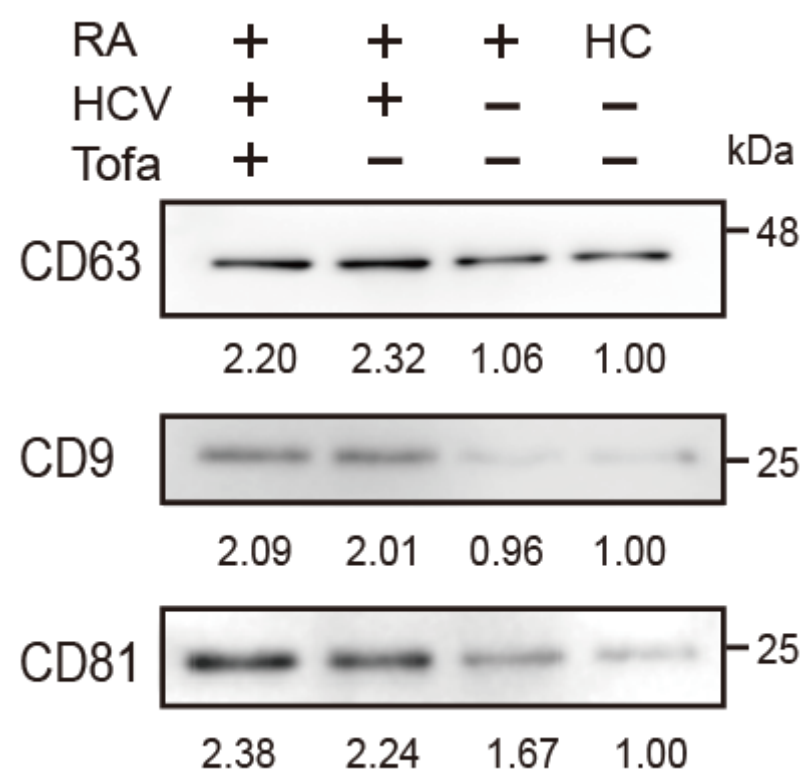

CD63 (43kDa)

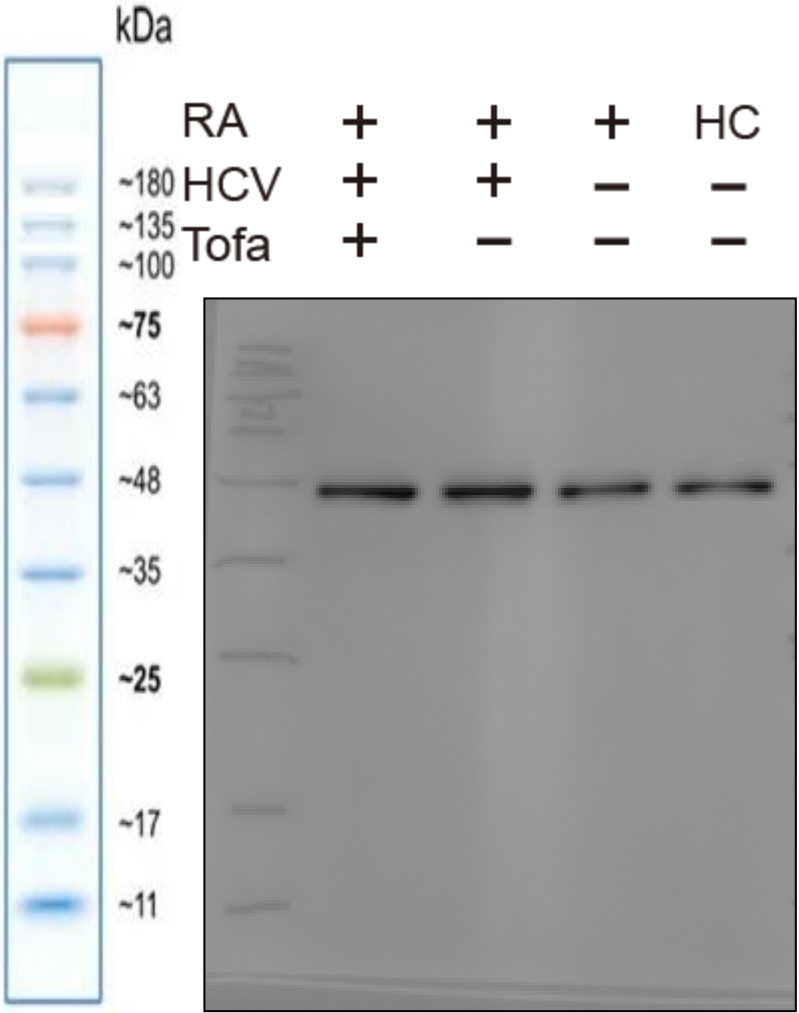

CD9 (24kDa)

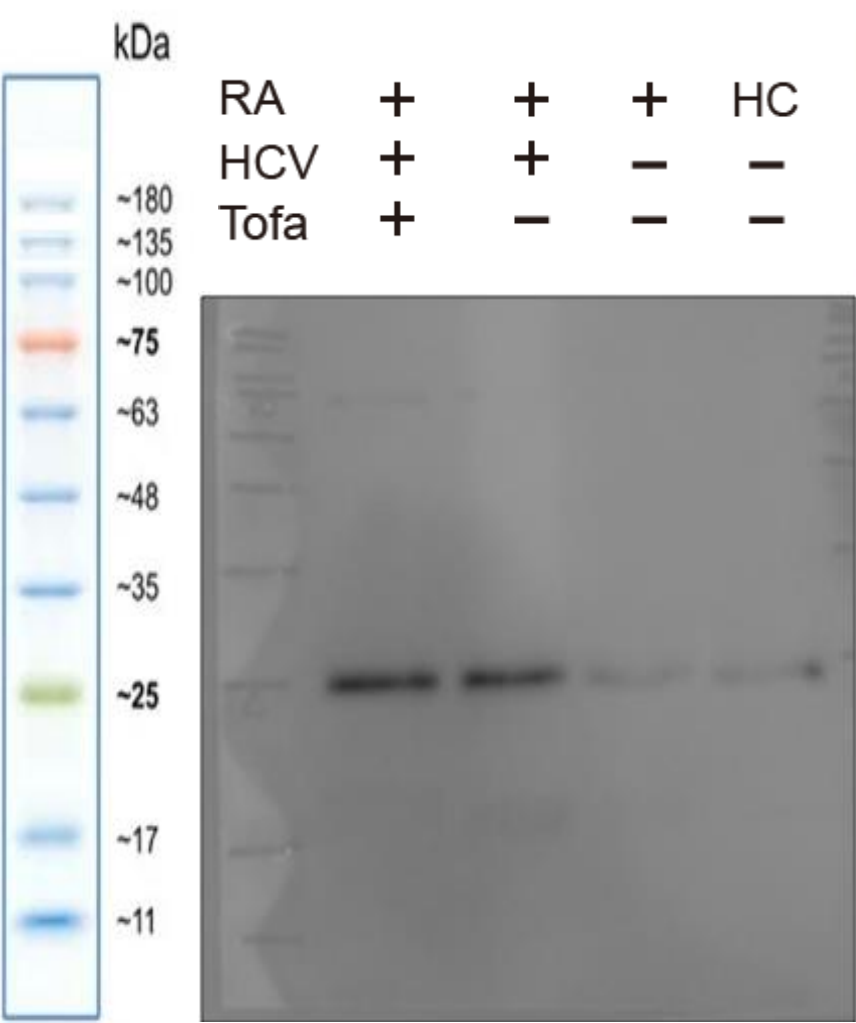

CD81 (25kDa)

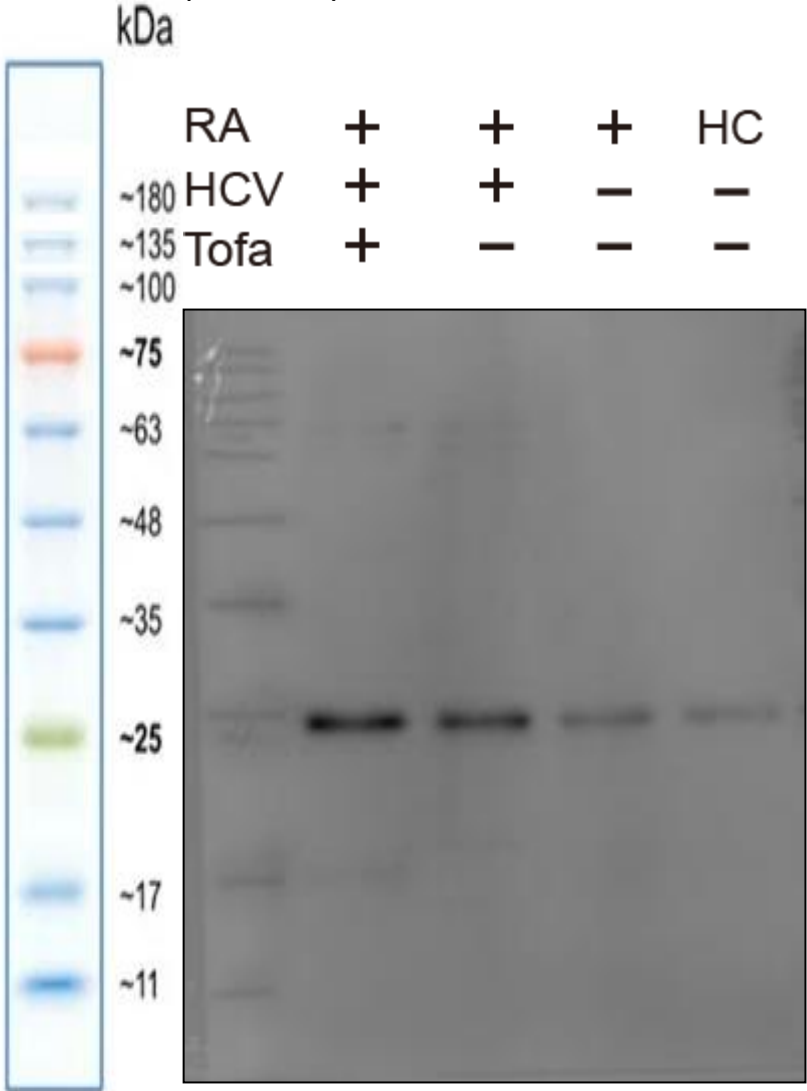

Supplement: Supplementary file 2 — Supplementary Figure S2. [file 41598_2023_50963_MOESM2_ESM.pdf]
